# Influence of Preoperative Peripheral Parenteral Nutrition with Micronutrients after Colorectal Cancer Patients

**DOI:** 10.1155/2015/535431

**Published:** 2015-04-27

**Authors:** Ming-Yi Liu, Hsiu-Chih Tang, Shu-Hui Hu, Hui-Lan Yang, Sue-Joan Chang

**Affiliations:** ^1^Department of Nutrition, Tainan Sin-Lau Hospital, No. 57, Sec. 1, Dongmen Road, Tainan 70142, Taiwan; ^2^Department of Surgery, Tainan Sin-Lau Hospital, No. 57, Sec. 1, Dongmen Road, Tainan 70142, Taiwan; ^3^Department of Medical Laboratory Science and Biotechnology, Kaohsiung Medical University, No. 100, Tzyou 1st Road, Kaohsiung 807, Taiwan; ^4^Department of Nutrition and Dietetics, Kaohsiung Medical University Hospital, No. 100, Tzyou 1st Road, Kaohsiung 807, Taiwan; ^5^Department of Nursing, Tainan Sin-Lau Hospital, No. 57, Sec. 1, Dongmen Road, Tainan 70142, Taiwan; ^6^Department of Life Sciences, College of Bioscience and Biotechnology, National Cheng Kung University, No. 1, University Road, Tainan 701, Taiwan

## Abstract

*Background*. The inflammatory reactions are stronger after surgery of malnourished preoperative patients. Many studies have shown vitamin and trace element deficiencies appear to affect the functioning of immune cells. Enteral nutrition is often inadequate for malnourished patients. Therefore, total parenteral nutrition (TPN) is considered an effective method for providing preoperative nutritional support. TPN needs a central vein catheter, and there are more risks associated with TPN. However, peripheral parenteral nutrition (PPN) often does not provide enough energy or nutrients. *Purpose*. This study investigated the inflammatory response and prognosis for patients receiving a modified form of PPN with added fat emulsion infusion, multiple vitamins (MTV), and trace elements (TE) to assess the feasibility of preoperative nutritional support. *Methods*. A cross-sectional design was used to compare the influence of PPN with or without adding MTV and TE on malnourished abdominal surgery patients. *Results*. Both preoperative groups received equal calories and protein, but due to the lack of micronutrients, patients in preoperative Group B exhibited higher inflammation, lower serum albumin levels, and higher anastomotic leak rates and also required prolonged hospital stays. *Conclusion*. Malnourished patients who receive micronutrient supplementation preoperatively have lower postoperative inflammatory responses and better prognoses. PPN with added fat emulsion, MTV, and TE provides valid and effective preoperative nutritional support.

## 1. Introduction

Patients with surgical stress face many challenges, including maintaining good nutritional status and avoiding weight loss. Preoperative nutrition support can improve patient tolerance of and outcomes for surgery, especially when provided to malnourished patients before major abdominal surgery [1]. Postoperative changes of cytokines such as interleukin-6, tumor necrosis factor, and interleukin-8 have been studied previously [2–6]. In addition, the relation of cytokines to polymorphonuclear elastase and C-reactive protein (CRP) levels has also been examined in the context of surgical invasion [2, 4, 7, 8]. A variety of cytokines play important roles in host responses; however, exaggerated systemic cytokine responses may be harmful to the host. Inflammation may alter metabolism, increase energy and protein requirements, and increase the risk of infection. Previous studies have shown that the inflammatory reactions after surgery of malnourished preoperative patients are greater than those of patients with good preoperative nutritional status [9, 10].

Many studies have shown the relationship between inflammation and micronutrients. Vitamin deficiencies appear to affect the functions of immune cells. A deficiency in vitamin D, for example, increases the risk of infectious and inflammatory diseases, while a vitamin A deficiency causes an increase in inflammatory responses. Vitamin E is deficiency associated with a defect of naive T cells [11]. Zinc deficiency influences the generation of cytokines, and in response to zinc supplementation, plasma cytokines exhibit a dose-dependent response [12]. In enhanced recovery programs, oral nutritional supplements (ONS) are administered to malnourished patients both before surgery and for at least the first 4 postoperative days. For significantly malnourished patients, elderly patients, and patients with chronic diseases, all of whom may have micronutrient deficiencies or ingest vitamins and minerals at rates below recommended doses and who thus may need supplementation before and after surgery, nutritional supplementation (oral and/or parenteral) has the greatest effect if started 7 to 10 days preoperatively [13, 14].

Enteral feedings are the preferred strategy for the delivery of such nutrition. However, enteral nutrition is often inadequate for very malnourished patients. Total parenteral nutrition (TPN) is a common method for providing preoperative nutritional support to compensate for the deficiency of enteral nutrition in severely malnourished patients facing abdominal surgery [15, 16]. TPN solutions contain fat emulsion, vitamins, and trace elements to support complete nutrition, but there are risks associated with receiving TPN, including pneumothorax, hemothorax, and central vein catheter infection. Peripheral parenteral nutrition (PPN) is easier to use and less risky than TPN; however, the PPN solutions that are generally used often do not provide enough energy and nutrients. Because more active nutritional support is essential to achieve complete nutrition and thereby improve patient tolerance and outcomes, modified PPN solutions have been tried and successfully applied [17]. Recently, combinations of two-in-one (dextrose + amino acids) formulas or fat emulsion with PPN have been commonly used. However, multiple vitamins (MTV) and trace elements (TE) are still often omitted from PPN formulas for short-term preoperative support. Clinical experience has demonstrated that trace elements cannot be added to PPN because of the resulting high osmolarity, which in most cases causes phlebitis of the peripheral vein.

It is suggested that preoperative TPN support be administered for 7 to 10 days. However, receiving more than 7 days of TPN support is inconvenient for some patients and has apparently made some patients in Taiwan reluctant to be hospitalized. The aim of this cross-sectional study was to investigate the influence on malnourished abdominal surgery patients of receiving short-term (4 to 5 days) PPN with or without adding MTV and TE. Accordingly, the results of the study can serve as a reference in assessing the feasibility of preoperative nutritional support.

## 2. Materials and Methods

### 2.1. Patients

In this study, a cross-sectional design was used. We screened a database of intensive care unit (ICU) patients at Tainan Sin-Lau hospital covering a period from 2010 to 2013. The inclusion criteria were as follows: patients with high preoperative malnutrition risk who had undergone laparoscopic surgery for colorectal cancer and received preoperative PPN support. However, end-stage renal disease patients in the preoperative stage and postoperative patients with short bowel syndrome were excluded from this study. The included patients were classified into two groups. Group A patients received preoperative PPN with fat emulsion, MTV, and TE. Group B patients received preoperative PPN with fat emulsion only. Acquisition of patient data and its subsequent use were approved by the ethics committee of the Tainan Sin-Lau Hospital (Grant number SLH919-02). Patient information was anonymized and deidentified prior to analysis.

### 2.2. Malnutrition Risk Screening

Malnutrition risk was assessed based on the malnutrition screening tool (MST) [18–20] and serum albumin levels. The MST is a quick and simple nutrition screening tool based on weight loss and appetite changes (Table 1). Subjects with a score of 2 or more and a serum albumin of below 3.5 g/dL were subsequently classified as being at risk of malnutrition.

### 2.3. Preoperative Nutrition Support

When a patient was recognized as having high malnutrition risk before surgery, we encouraged the patient to eat by oral or enteral feeding as tolerated in order to supply nutrition and maintain the integrity of the intestinal mucosa. When a patient consumed less than 18 kcal per kilogram of body weight by enteral feeding, then the PPN intervention was considered. Patients received PPN for 5 days before the operation. The PPN consisted of two-in-one formulas (Clinimix, Baxter International, Inc., United Kingdom) (Table 2), with both patient groups receiving 1500 mL of PPN solution and 200 mL of a 20% fat emulsion (Lipofundin, B Braun Ltd., Melsungen, Germany) daily. The total calorie count of the formulas was 996 kcal, and they also contained 42 grams of protein and 40 grams of fat. The formula for Group A patients also had added MTV (Infuvita, Yu-Liang Pharmaceuticals Co., Taiwan) and TE (Trace Element Injection, China Chemical & Pharmaceutical Co., Ltd., Taiwan). Each patient received 3.0 mg of zinc per day to promote postoperative wound healing (Table 3). The PPN was delivered through an intravenous line; in order to avoid phlebitis, the injection site was changed every three days.

### 2.4. Postoperative Nutrition Support

TPN support was performed immediately for patients in both groups if the given patient was expected to be unable to obtain nutrients from the gut beyond the first five days postoperatively. Moreover, TPN support was given when the gut became tolerant of combined enteral nutrition support. The TPN formulations were varied according to individual patient nutritional needs. If the given patient could tolerate enteral nutrition, we turned a given TPN formula into a PPN formula (Clinimix, Baxter International Inc.). Patients in both groups were treated according to the same follow-up protocol; specifically, each patient received 18 kcal per kilogram of body weight on postoperative day- (POD-) 1, 23 kcal on POD-3, and 28 kcal on POD-7. We gradually tapered the amount of TPN as the patients became tolerant of enteral nutrition. Parenteral nutritional support was suspended until a patient could take in 70% of his or her nutritional goal via the gut.

### 2.5. Data Collection

Blood was collected before the operation and on POD-1, POD-3, and POD-7. Albumin, white blood cell (WBC), and C-reactive protein (CRP) levels were measured. Vitamin D3 (25-OH) and zinc concentrations were checked on POD-1. The sepsis-related organ failure assessment (SOFA) score was calculated and postoperative hospital days, operative time, blood loss, and any complications were recorded.

### 2.6. Statistical Analysis

Data were analyzed using SPSS version 12.0 (SPSS, Inc.). The differences in nutritional statuses between the two groups were analyzed by Student's *t*-test. Data are presented as means ± SD. Mortality analyses were performed using chi-square analysis. A *P* value of <0.05 was taken to indicate a significant difference.

## 3. Results

### 3.1. Preoperative Patient Characteristics

Data for 121 cases were collected and divided into 2 groups; the patient characteristics are provided in Table 4. No significant differences between the two groups were found for age, MST score, and number of preoperative days during which PPN was received (PPN days). The preoperative serum albumin levels of both groups were 3.18 ± 0.2 and 3.13 ± 0.2 mg/dL, respectively (*P* = 0.180). Both groups received 1500 mL PPN solution for preoperative nutritional support. Patients were also encouraged to take nutrition orally as tolerated, and the average intake was 735.6 ± 246.3 kcal per day. There was no significant difference in the total daily calories and protein (PPN + oral intake) per kilogram of body weight for the two patient groups (Table 4). The average for total calories obtained was about 29 kcal/kg, while the average protein intake approached 1.2 g/kg.

### 3.2. Postoperative Nutritional Support

TPN support was performed immediately for patients in both groups depending on whether the patient was hemodynamically stable. There was no significant difference in the total calories and protein levels for the two groups (Table 5). Both groups of patients achieved intake of 25 kcal and 1.2 grams of protein per kilogram of body as of POD-7.

### 3.3. Postoperative Nutrition Conditions and Outcomes

There was no significant difference between the two groups in terms of the operative time and estimated intraoperative blood loss (Table 5). For both groups, postoperative serum albumin levels were obviously decreased compared to the preoperative levels. The postoperative serum albumin on POT-1, POT-3, and POT-7 for Group A was higher than those for Group B (*P* < 0.05, Table 5). The WBC and CRP levels were significantly higher for Group B than for Group A (*P* < 0.001). Both groups received the same postoperative nutritional care, with no significant difference in the total calories and proteins per kilogram of body weight for the two groups. The above data implied that Group A patients had a higher average postoperative serum albumin level and lower postoperative inflammatory response. Three patients in Group A and four patients in Group B died during hospitalization; there was no significant difference between the two groups' mortality (3.9% versus 8.9% *P* = 0.260). The Group A patients exhibited a lower trend of SOFA scores than did the Group B patients, but there was no statistically significant difference (3.9 ± 1.3 versus 4.3 ± 1.2 *P* = 0.062). The rates of phlebitis and infectious complications among the two groups were similar, but the anastomotic leak rate for Group B patients was obviously higher than the rate for Group A patients (*P* = 0.026). We checked serum vitamin D3 (25-OH) and zinc concentrations on POD-1 and found that the Group A levels were better than those for Group B (*P* = 0.05). The postoperative hospital days showed that the Group B patients required significantly longer stays than did the Group A patients (11.26 ± 3.06 versus 14.96 ± 2.42 *P* < 0.001). These data indicated that the Group B patients had higher rates of infection and inflammation and more prolonged hospital stays.

## 4. Discussion

Our previous studies showed that the PPN with fat emulsion and micronutrients are convenient and effective nutritional support methods for surgical patients [17]. The extension studies suggested that supplying micronutrients can reduce postoperative inflammatory response for patients with preoperative malnutrition. We confirmed that the use of PPN with added fat emulsion, MTV, and TE for preoperative nutritional support was feasible and convenient. Although there were no significant differences in the mortality and the SOFA scores of the two groups, the inflammatory response rate and the postoperative hospital days were significantly reduced in Group A, meaning that the use of PPN with added fat emulsion, MTV, and TE could be beneficial for hospitals in terms of improving patient care quality and lowering costs. This study showed that administration of preoperative PPN with added fat emulsion, MTV, and TE for about 4 days was sufficient to achieve significant improvement in the prognosis. As such, this approach could be used to shorten the preoperative nutritional support period, which could be especially meaningful in dealing with emergency medical conditions.

Patients who are malnourished before surgery may lack various nutrients, which can cause adverse effects after surgery. Providing additional calories and protein via PPN with added MTV and TE can be beneficial to such patients. Many nutrients are obviously linked to wound healing. Zinc is an important trace mineral for DNA synthesis, cell division, and protein synthesis. The crucial role of zinc is well documented and zinc deficiency delays wound healing [21]. Zinc deficiency often occurs in surgical patients. Preoperative PPN support with added zinc may improve zinc storage to cope with stress after surgery. Both groups received equal calories and protein preoperatively, but Group B patients exhibited higher inflammation and delayed wound healing due to the lack of micronutrients. WBC and CRP levels are common markers of infection or inflammation. CRP is a nonspecific marker of inflammation, and the measurement of CRP after colorectal surgery can predict the risk of adverse events and a prolonged hospital stay [22]. In this study, the Group B patients, who had a higher average CRP level that may have been caused by their higher anastomotic leak and infection rates, also required longer periods of postoperative hospitalization.

Enteral nutrition can stimulate hormone secretion, promote the portal circulation, and maintain the barrier and immune function of the intestinal mucosa [23]. However, enteral nutritional support before major abdominal surgery is often insufficient due to gut dysfunction. Therefore, enteral support combined with parenteral nutrition support is often considered. TPN is a commonly used method of nutritional support to compensate for the deficiency of enteral nutrition. In past studies, it has been suggested that preoperative TPN support be administered for 7 to 10 days. However, TPN requires a central venous catheter, which entails risks and inconvenience for patients. Traditionally, PPN formulas included glucose, amino acids, and electrolytes but were inadequate in terms of vitamins and trace elements, and these formulas were only used for short-term (5 to 8 days) support. A modified PPN formula could improve on these problems, especially if MTV and TE are added. We thought that micronutrient deficiency is often a problem for patients with a gastrointestinal disease when they will undergo an operation. Although we provided only about 5 days of PPN support, there were obvious benefits for the Group A patients. Our PPN formula has the potential side effect of phlebitis as a result of the high osmolarity liquid (approximately 845 mOsm/L) injections to the peripheral vein wall. We changed the injection site every three days for patients and found that keeping the mechanical interference at the injection site to a minimum can effectively decrease the occurrence of phlebitis. Nonetheless, some patients still complained of pain during the implementation of preoperative PPN. Therefore, determining how to avoid phlebitis is another challenge for preoperative PPN.

The main limitation of this study consisted of the retrospective study design. We did not check the postoperative changes of cytokines such as interleukin-6, tumor necrosis factor, and interleukin-8. The fat emulsion which we used was a 20% medium-chain triglyceride/long-chain triglyceride (MCT/LCT) emulsion. Recently, studies have shown that supplementation of *ω*-3 fatty acids in PPN may improve the inflammatory response and immune response [24]. Therefore, it will be worth exploring whether our PPN formula combined with a *ω*-3 fatty acid emulsion would be more beneficial for surgical patients. Potential prospective studies could include comparisons of the influence of preoperative standard formulas and immunonutrition parenteral nutrition formulas.

## 5. Conclusion

Patients with a good preoperative micronutrient status have lower postoperative inflammatory responses. PPN with added fat emulsion, MTV, and TE provides valid and effective preoperative nutritional support.

## Figures and Tables

**Table 1:** Malnutrition screening tool (MST).

(1) Has the resident/patient lost weight recently without trying?	
No	0
Unsure	2
Yes, how much?	
1–5 kg	1
6–10 kg	2
11–15 kg	3
>15 kg	4
(2) Has the resident/patient been eating poorly because of a decreased appetite?	
No	0
Yes	1

	Total score_______

MST based on weight loss and appetite changes. Subjects with a score of 2 or more were identified as being at risk of malnutrition.

**Table 2:** Ingredients of per liter peripheral parenteral nutrition formulations.

Glucose (%)	Amino acid (%)	Na mEq/L	K mEq/L	Cl mEq/L	Mg mEq/L	Ca mEq/L	P mM/L	Acetate mEq/L	kcal/L
7.5	2.8	35	30	40	2.5	2.3	15	50	410

Other nutrients added: Infuvita injection 10 mL/day; trace elements: 2 mL/day; 20% MCT/LCT fat emulsion (100 mL/Bot.) 2 Bot./day.

**Table 3:** The ingredients of vitamins and minerals added to the 1500 mL PPN solution.

Infuvita 10 mL	Trace elements 2 mL
Ascorbic acid 100 mg	Zn 3.0 mg
Vitamin A 3300 IU	Copper 1.0 mg
Vitamin D 200 IU	Manganese 0.4 mg
Thiamin 3 mg	Chromium 0.01 mcq
Riboflavin 3.6 mg	Iodine 0.056 mcq
Pyridoxine 4 mg	
Niacin 40 mg	
Pantothenic acid 15 mg	
Vitamin E 10 IU	
Biotin 60 mcg	
Folic acid 400 mcg	
Vitamin B12 5 mcg	

**Table 4:** Preoperative nutritional status for two groups of patients.

	Group A	Group B	*P*
*n*	76	45	
Gender (M/F)	41/35	29/16	
Age	68.9 ± 9.8	66.4 ± 10.0	0.186
MST score	2.67 ± 0.7	2.64 ± 0.6	0.832
Serum albumin (g/dL)	3.18 ± 0.2	3.13 ± 0.2	0.093
PPN days	5.2 ± 0.67	5.3 ± 0.7	0.184
Total calories (kcal/kg)	28.5 ± 2.4	29.3 ± 3.1	0.306
Total protein (g/kg)	1.2 ± 0.14	1.1 ± 0.15	0.370

For the two groups, no significant differences were found with regard to age, MST score, serum albumin, and number of preoperative days during which PPN was received (PPN days).

Values are presented as number of patients or mean ± SD.

MST: malnutrition screening tool.

PPN: peripheral parenteral nutrition.

*P* < 0.05.

**Table 5:** Postoperative nutritional status for the two groups of patients.

	Group A	Group B	*P*
Albumin (mg/dL)			
POD-1	2.73 ± 0.3	2.67 ± 0.26	0.025^∗^
POD-3	2.70 ± 0.29	2.52 ± 0.25	<0.001^∗∗^
POD-7	3.19 ± 0.18	3.09 ± 0.19	0.007^∗^
WBC (1000/uL)			
POD-1	12.45 ± 2.34	14.76 ± 1.85	<0.001^∗∗^
POD-3	13.56 ± 2.0	16.12 ± 1.59	<0.001^∗∗^
POD-7	10.79 ± 2.26	12.94 ± 1.89	<0.001^∗∗^
CRP mg/L			
POD-1	53.28 ± 21.04	87.88 ± 37.14	<0.001^∗∗^
POD-3	59.2 ± 22.04	97.9 ± 39.9	<0.001^∗∗^
POD-7	19.1 ± 6.54	26.1 ± 6.99	<0.001^∗∗^
Postoperative hospital days	11.26 ± 3.06	14.96 ± 2.42	<0.001^∗∗^
SOFA score	3.9 ± 1.3	4.3 ± 1.2	0.062
Operative time (min)	197.3 ± 29.1	192.3 ± 20.4	0.497
Estimated blood loss (mL)	371 ± 55	367 ± 44	0.838
In-hospital mortality (%)	3.9	8.9	0.260
Phlebitis rate (%)	28.9%	24.4	0.590
Infectious complications (%)	11.8	22.2	0.129
Anastomotic leaks (%)	6.6	20	0.026^∗^
Vitamin D3 (25-OH) (ng/mL)	47.1 ± 7.4	38.0 ± 7.1	<0.001^∗∗^
Zinc (*μ*g/L)	1087 ± 120	982 ± 132	0.024^∗^
Total calories (kcal/kg)			
POD-1	14.7 ± 2.5	13.4 ± 2.6	0.109
POD-3	20.6 ± 2.0	20.1 ± 2.3	0.510
POD-7	25.6 ± 1.4	26.1 ± 1.7	0.222
Total protein (g/kg)			
POD-1	0.95 ± 0.1	0.95 ± 0.09	0.856
POD-3	1.16 ± 0.07	1.17 ± 0.07	0.530
POD-7	1.26 ± 0.06	1.27 ± 0.11	0.550

Vitamin D3 (25-OH) and zinc concentration were measured on POD-1.

Values are presented as number of percentage or mean ± SD.

POD: postoperative day.

SOFA: sepsis-related organ failure assessment.

WBC: white blood cell.

CRP: C-reactive protein.

^∗^ *P* < 0.05.

^∗∗^ *P* < 0.001.
